# High Accuracy of Restoration of the Individual Hip Anatomy Using Custom-Made Prostheses in Total Hip Arthroplasty

**DOI:** 10.3390/jcm14062115

**Published:** 2025-03-20

**Authors:** Maximilian F. Kasparek, Anna Jungwirth-Weinberger, Kirubakaran Pattabiraman, Marios Loucas, Devanand Hulmani, Maximilian Muellner, Tobias Scheidl, Oliver Haider, Thomas Muellner

**Affiliations:** 1 Department of Orthopedic Surgery and Traumatology, Evangelisches Krankenhaus, 1180 Vienna, Austria; a.jungwirth@ekhwien.at (A.J.-W.); tobias.scheidl@modul.at (T.S.);; 2 Department of Orthopedics, AIIMS, Sri Aurobindo Marg, Ansari Nagar, Ansari Nagar East, New Delhi 110029, India; kiru2209@gmail.com; 3 Trustwell Hospitals Pvt. Ltd., Vinobha Nagar, Sudhama Nagar, Bengaluru 560002, India; devanand.hulmani@gmail.com; 4 Center for Musculoskeletal Surgery, Charité—Universitätsmedizin Berlin, Klinik für Orthopädie, Schumannstraße 20, 10117 Berlin, Germany; maximilian.muellner@charite.de

**Keywords:** total hip arthroplasty, custom-made prosthesis, femoral offset, femoral anteversion, acetabular inclination, acetabular anteversion

## Abstract

**Background/Objectives**: Femoral or acetabular deformities are important factors in development of early osteoarthritis. In particular, young patients benefit from individual anatomical restoration by decreasing the risk of early loosening and wear and achieving a good clinical outcome. **Methods**: This prospective study evaluates the use of a custom-made prosthesis in anterior approach total hip arthroplasty (THA). Pre- and postoperative imaging included conventional X-rays as well as computer tomography (CT) with a specialized protocol to analyze femoral diaphysis width, horizontal and vertical offset, caput-collum-diaphyseal (CCD) angle, leg length, femoral and acetabular anteversion angles, and the position of the center of rotation. **Results**: A total of 22 hips (11 female, 11 male) with a mean age of 55.8 years underwent THA with a custom-made prosthesis (Symbios^®^). Accurate restoration has been shown for offset, leg length, and femoral anteversion. The custom stems showed a good fit within the femoral canal. **Conclusions**: This custom-made prosthesis has been shown to be a valuable option for the treatment of hip osteoarthritis in young patients, with adequate restoration of the preoperative anatomy.

## 1. Introduction

Hip pain is a prevalent issue that significantly restricts daily activities, leading to reduced quality of life and contributing to various comorbidities [1]. The etiology of hip pain is diverse, encompassing conditions such as avascular necrosis of the hip (AVN), inflammatory arthritis, infective arthritis, developmental dysplasia of the hip (DDH), and primary degenerative arthritis [2]. The causes of hip pain, necessitating hip replacement surgery, vary across different age groups. In younger patients, conditions such as femoroacetabular impingement (FAI), DDH, sequelae of infection, slipped capital femoral epiphysis (SCFE), and trauma are common culprits that lead to early degeneration of the hip joint [3,4,5,6,7]. Additionally, AVN is frequently encountered in younger individuals, often associated with risk factors such as alcohol or steroid abuse [8].

Variation in the proximal femoral and acetabular anatomy plays a crucial role in the development of early degenerative changes in the hip joint. In contrast, older patients commonly suffer from primary degenerative arthritis, necessitating a different clinical approach to hip replacement. The evolution of THA over the decades has made it one of the most successful and frequently performed orthopedic procedures, often referred to as the “Orthopedic Operation of the Century” [9]. The primary goal of THA is to restore the individual geometry of the patient’s hip, which is crucial for minimizing wear and maximizing the longevity of the implant [10]. Achieving this goal involves selecting the most appropriate implant system and surgical approach, tailored to the specific needs of each patient.

Significant advancements in hip arthroplasty have introduced better components and implant systems, which have made THA a viable option even for younger patients [10]. However, performing THA in younger patients presents unique challenges due to their higher functional demands and longer life expectancy [11]. These patients often present with anatomical deformities in the hip due to conditions such as dysplasia, posttraumatic arthritis, and sequelae of infection. Such deformities predispose them to complications such as aseptic loosening of the prosthesis due to micro-movements, incomplete “fit and fill”, and exposure to excessive forces resulting from a more active lifestyle [12,13].

Cementless femoral stems, while advantageous in many cases, have limitations when used in younger patients with altered proximal femoral anatomy. These stems, designed with fixed extra- and intramedullary dimensions, may not accommodate the anatomical variations seen in these patients, leading to increased risks of intra-operative complications such as periprosthetic fractures, proximal stress shielding, and impingement [14,15]. Ensuring the long-term success of a hip replacement in younger patients requires primary stem stability, which promotes proper bone ingrowth or ongrowth, leading to durable fixation [16]. This stability is highly dependent on the accurate filling of the proximal femur and the correct positioning of the femoral stem [17,18].

Anatomical variations in the femoral canal and extramedullary parameters between patients with dysplastic hips and those with primary osteoarthritis further complicate the fitting of conventional femoral stems [19]. Intramedullary variations, such as proximal femur deformities, increased canal flare index, lateral curvature, and femur helitorsion, create significant challenges during hip replacement surgery [20]. Extramedullary factors, including acetabular and femoral offset, femoral neck length and angle of anteversion, and femoral neck angle (FNA), are equally critical, as they influence the function of the abductor musculature [21].

In addition to intramedullary variations, the extramedullary parameters of the proximal femur also differ significantly, particularly in cases with inadequate or excessive acetabular coverage [22]. Traditional 2D imaging techniques, such as anteroposterior radiographs, often fail to account for anteversion and external rotation, leading to measurement errors in femoral offset that can occur in approximately 40% of cases [23]. These errors can result in significant clinical issues, such as abductor weakness or decreased abductor function [24].

A common issue with conventional stems is that they are designed to fit intramedullary, often creating a new center of rotation that does not match the original anatomical center of the femoral head [25]. This mismatch can lead to suboptimal functional outcomes, particularly in cases where the femoral stem does not fit well in the metaphyseal zone [13].

Given the anatomical challenges and the limitations of conventional THA, the use of customized hip prostheses has become increasingly relevant. Customized prostheses are designed to account for the unique anatomical features of each patient, ensuring an optimal fit that restores the natural hip anatomy as closely as possible. This approach is particularly beneficial in restoring the center of rotation and achieving the correct positioning of the femoral stem, which are essential for successful outcomes in hip replacement surgery [26,27]. The present study therefore aims to determine how accurately the native geometry of the hip is restored when custom-made hip prostheses are used.

## 2. Materials and Methods

This retrospective study included a consecutive series of 22 cases, in which patients received a femoral custom-made THA. Ethical approval by the local institution board was obtained prior to the study (EK Nr. 11/2023).

### 2.1. Custom-Made Prosthesis Preparation

The custom-made hip prostheses were procured from SYMBIOS^®^ (Yverdon-les-Bain, Switzerland). Each prosthesis was designed following meticulous preoperative planning and the approval of the operating surgeon.

Preoperative planning was conducted by the company using both X-rays and CT scans to assess critical parameters. X-ray assessments included measurements of femoral diaphysis width, horizontal and vertical offsets, caput-collum-diaphyseal (CCD) angle, and leg length. CT scans provided detailed assessments of leg length, femoral and acetabular anteversion angles, and the center of rotation position.

**Figure 1:**
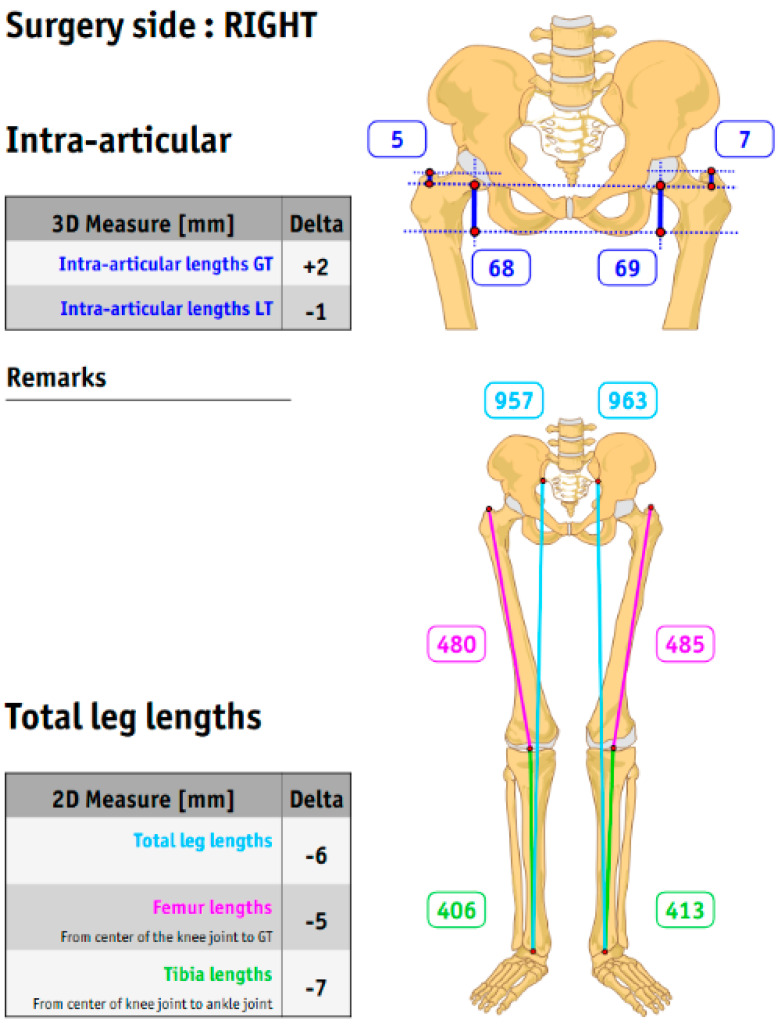
Preoperative intraarticular and leg length measurement, considering the contralateral side and leg length discrepancy.

Intraarticular leg length discrepancies were assessed using three-dimensional measurements. The distances from the greater trochanter (GT) and lesser trochanter (LT) to the center of rotation were analyzed. Additionally, the total leg length, as well as the lengths of the femur and tibia, were measured and are presented in Figure 1.

All patients underwent the same protocol postoperatively and were evaluated by the company.

### 2.2. CT Imaging Protocol

CT images were obtained using a protocol specifically designed to be compatible with the HIP-PLAN^®^ 3D hip planning software. This software, when used in conjunction with SYMBIOS standard hip implants, allowed for increased accuracy in surgical planning compared to conventional X-ray templating. The CT imaging protocol differed from standard diagnostic imaging protocols, focusing on precise measurements required for custom prosthesis design.

**Figure 2:**
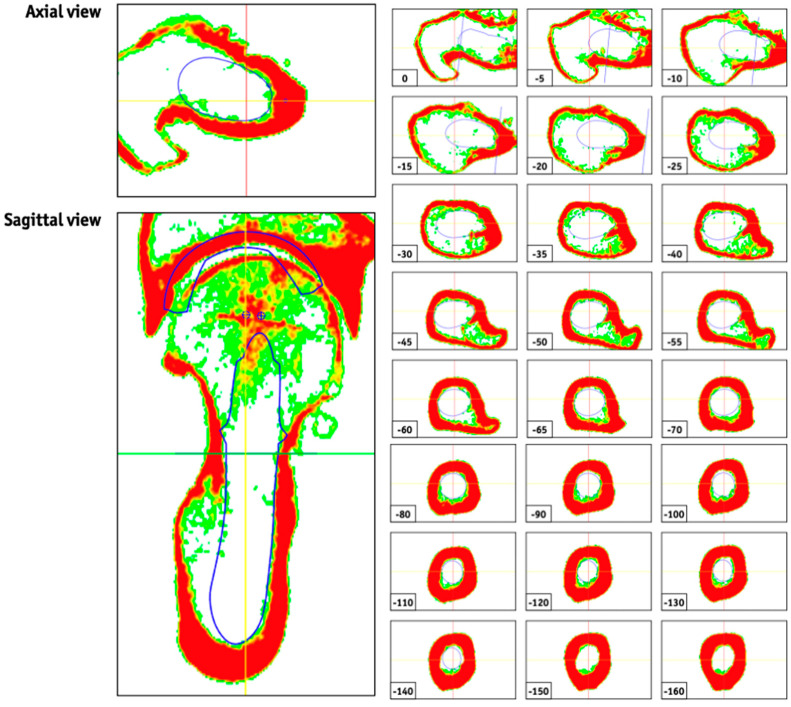
CT-based topograms illustrating the planned stem position in axial and sagittal views along the diaphyseal course.

Patients were positioned supine with feet pointing forward, and legs extended and aligned with the table’s axis. In order to ensure comfort and minimize movement during the examination, cushions and straps were used as necessary. Scout views (topograms) were performed with the highest possible resolution to calibrate reconstruction parameters, both axial and sagittal. An example is presented in Figure 2, illustrating the complete diaphyseal course of the planned position of the custom-made stem.

The CT protocol involved obtaining 5 mm cuts from the acetabulum to the greater trochanter and 10 mm cuts from the lesser trochanter to the femoral isthmus, facilitating precise intramedullary reconstruction of the femur. Anteversion was calculated using horizontal cuts at the level of the 2nd metatarsal, femoral condyles, and 10 mm above the lesser trochanter. This protocol also enabled the calculation of femoral neck angle (FNA) and femoral anteversion, essential for custom stem design.

### 2.3. Preoperative Planning and Custom Stem Design

Custom stem design was based on detailed preoperative X-ray images and the CT protocol. Three-dimensional reconstruction of the femoral canal was necessary to prevent over-reaming of cancellous bone and to ensure cortical contact of the stem. The metaphyseal cancellous bone was impacted before stem implantation using custom-designed rasps that matched the stem dimensions. This protocol allowed for the correction of extramedullary parameters, including offset and neck anteversion.

**Figure 3:**
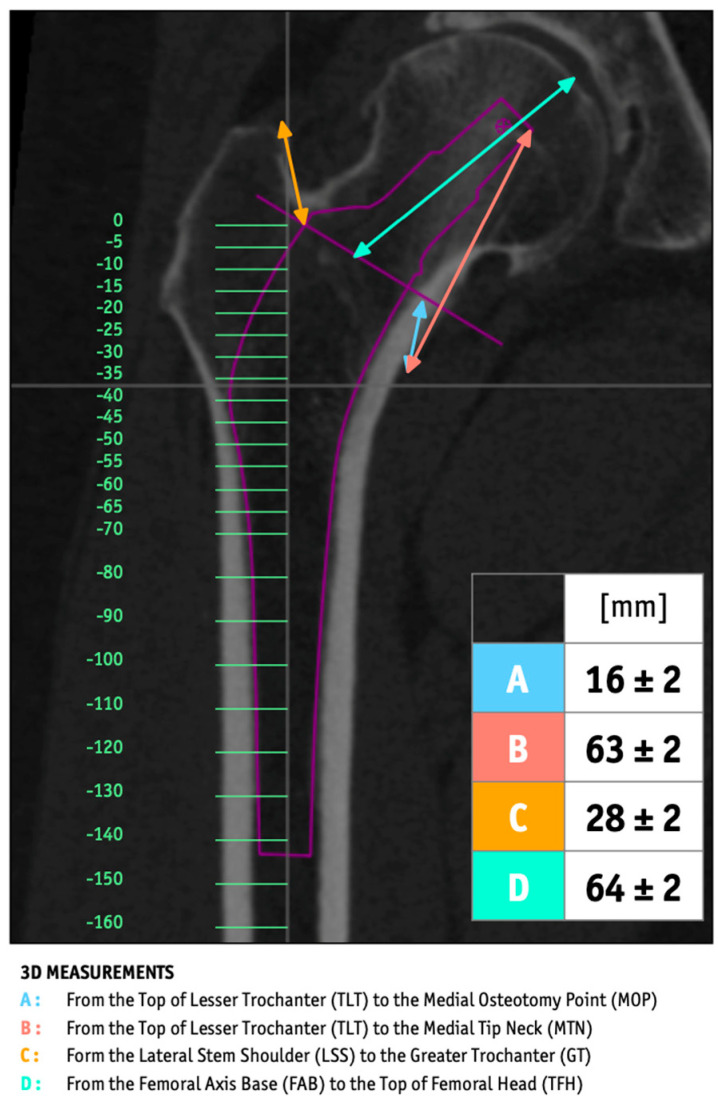
Illustration of the Stem Planning Face.

During preoperative planning, actual-size (1:1) planning was used in the operating room, termed “Face-Osteo” planning. This approach provided precise bone resection parameters and CT level calibration, ensuring accurate stem implantation. Various measurements were taken, including distances from the lesser trochanter to the medial neck, the lateral stem curve to the greater trochanter, and the femoral axis to the top of the femoral head, among others. These measurements are illustrated in Figure 3.

CT reconstruction of bone density in the metaphyseal-diaphyseal area with the stem in situ allowed for the assessment of cancellous bone density, visualization of necessary bone mass removal, and control of the correct stem position at the level of the osteotomy.

### 2.4. Surgical Technique

All THA procedures were performed using a direct anterior approach with an extension table by an experienced arthroplasty surgeon (TM). The incision was made three cm distal and three cm lateral to the anterior superior iliac spine (ASIS) and extended distally and laterally over the tensor fasciae latae (TFL) muscle. The fascia of the TFL was split, and blunt dissection was performed to expose the femoral neck. After ligating the ascending branch of the lateral femoral circumflex artery, a capsulotomy was performed, and the femoral neck was cut in situ with an oscillating saw. The femoral head was removed using a corkscrew after making two cuts in the femoral neck.

**Figure 4:**
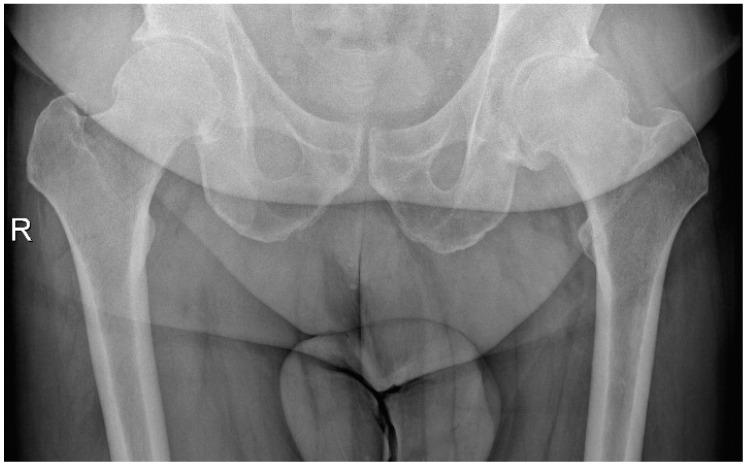
Male patient (53 years old) with coxarthritis of the left hip.

**Figure 5:**
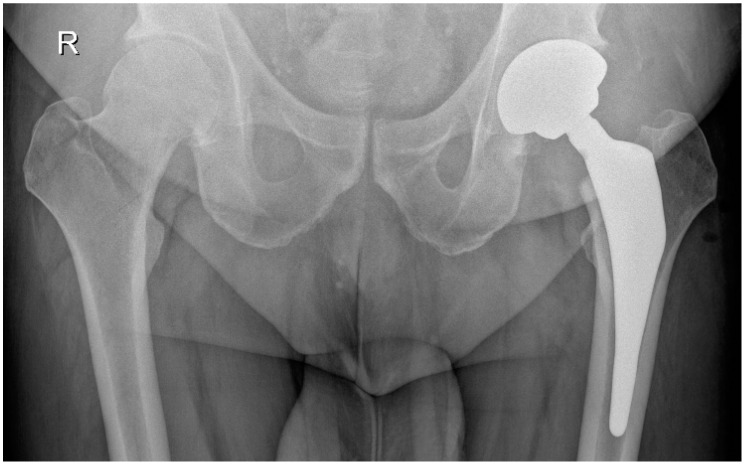
Postoperative X-ray of the same patient after a custom-made THA on the left side.

Acetabular reaming and cup placement were performed using offset reamers and cup insertion handles. After a capsular release performed with electrocautery, the femur was prepared using a single custom-made rasp. The custom-made femoral stem, with 2/3 hydroxyapatite coverage and ceramic femoral heads, was implanted, and hip stability was checked post-reduction. Figure 4 and Figure 5 show a patient with coxarthritis of the left hip and his postoperative X-ray.

After local infiltration anesthesia was administered, the capsule and TFL fascia were closed with stitches. The wound was then closed using subcutaneous sutures and an intradermal skin suture, and finally, steristrips and a plaster were applied.

### 2.5. Postoperative Assessment and Rehabilitation

A postoperative CT scan was performed to evaluate the accuracy of implant placement and restoration of the center of hip rotation. The rehabilitation protocol included immediate full weight bearing, depending on the patient’s tolerance. Standard antithrombotic treatment with low-molecular-weight heparin was administered for 35–40 days. In one patient, postoperative CT scans revealed an acetabular rim fracture and a dislocated inlay. This patient was promptly scheduled for revision surgery, during which the components were exchanged for an IMPLANTEC^®^ Ana Nova hybrid cup with a ceramic inlay.

### 2.6. Statistical Analysis

The statistical analysis was conducted using IBM SPSS Statistics, version 29.0.2.0 (IBM, Armonk, NY, USA). The distribution of all variables was assessed using the Shapiro–Wilk test. A paired samples t-test or Wilcoxon signed-rank test were used adequately for the comparison of pre- and postoperative data. For descriptive purposes, quantitative variables are expressed as mean ± SD and range. A *p*-value < 0.05 was considered statistically significant.

## 3. Results

The study included a consecutive series of 22 cases, with 11 female and 11 male patients who had a THA between November 2016 and March 2018. The average age of the patients at time of surgery was 55.8 ± 20.5 years (range 40–74).

Postoperative measurements showed a cup inclination ranging from 30° to 48°, with an average of 40.0° and an anteversion of 19.9°. The hip height, measured from the greater trochanter to the center of the hip, increased from 52.4 mm to 57.4 mm postoperatively (*p* < 0.001). Postoperatively, the average femoral offset increased from 39.7 mm to 46.2 mm, reflecting a lateralization of 6.5 mm (*p* < 0.001).

The mean femoral anteversion showed a slight decrease, from 16.8° preoperatively to 15.0° postoperatively (*p* = 0.112). Detailed information can be found in Table 1.

Prior to undergoing the surgical procedure, the affected limb exhibited an average reduction in length of 5.6 mm in comparison with the contralateral limb. After the surgical intervention, this discrepancy showed a substantial reduction, reaching an average of −0.2 mm (*p* = 0.003). An overview of the values concerning limb length discrepancy can be found in Table 2.

The custom-made femoral stems demonstrated a good fit within the femoral canal in all directions, maintaining a distance of less than 2 mm from the cortex. A detailed analysis of the stem fit can be found in Table 3.

One complication occurred in our cohort: one patient presented with a perioperative acetabular fracture and liner dislocation, and was treated with an exchange of the cup, liner, and head.

## 4. Discussion

Our study demonstrates the effectiveness of a custom-made Symbios^®^ prosthesis in restoring the patient’s anatomical structure postoperatively. These findings indicate that custom-made implants are a viable option for THA, particularly in younger patients, as the custom implants provide an accurate restoration of each patient’s anatomical characteristics, thereby reducing liner wear and maximizing the longevity of the implant [10].

Failure to restore the individual anatomy accurately can result in complications such as joint instability, leg length discrepancies, and altered gait mechanics, which can significantly impact the patient’s quality of life and the long-term success of the implant [4,12].

Traditional THA approaches often rely on standardized implants, which may not adequately address the unique anatomical challenges presented by each patient, as is possible with custom-made implants. This is particularly true for patients with complex hip deformities or atypical anatomical structures, where conventional prosthetic systems may not provide an optimal fit, leading to suboptimal outcomes [1,2].

Studies estimate that approximately 4–5% of patients requiring THA present with atypical anatomy, due to DDH and CDH. These patients often present unique challenges that standard implants cannot adequately address. For instance, younger patients with deformed femoral structures due to congenital or developmental conditions may require a more tailored approach to achieve the desired clinical outcomes. Custom hip prostheses have therefore emerged as a particularly valuable option for patients with atypical or complex hip anatomy that cannot be fully reconstructed using conventional implants.

This has led to the growing acceptance of the idea that every patient should be considered for custom hip implants, regardless of their age, gender, or underlying condition, to ensure the restoration of preoperative geometry [17].

Moreover, the study demonstrated significant improvements in femoral offset and limb length discrepancy postoperatively, highlighting the ability of custom prostheses to enhance hip biomechanics. These improvements are critical for reducing the risk of postoperative complications and ensuring a better overall functional outcome for patients [10].

The minor adjustment observed in femoral anteversion, with a decrease from 16.8 degrees preoperatively to 15.1 degrees postoperatively, is a significant finding in the context of joint stability and biomechanics. Femoral anteversion is crucial for proper joint alignment, and even slight deviations can lead to altered biomechanics and increased risk of complications. The fact that the custom prosthesis managed to maintain this alignment with only a −1.7 degree change suggests that it was effective in preserving the native hip anatomy. Mahmood et al. and Rüdiger et al. [23,24] emphasized that inadequate restoration of femoral anteversion can lead to abductor muscle weakness and increased joint reaction forces, potentially causing discomfort and impaired mobility. Therefore, the ability of the custom prosthesis to maintain near-native femoral anteversion is essential for reducing these risks and improving patient outcomes.

Moreover, the precise restoration of femoral anteversion is also critical in preventing impingement and dislocation, which are significant concerns in THA. As highlighted by Tsai et al. and Kim et al. [26,28], proper anteversion alignment minimizes the chances of postoperative complications, contributing to the long-term success of the implant. The small change observed in this study reinforces the importance of custom implants in achieving optimal alignment and stability, which are key to the overall success and longevity of the prosthetic hip joint. The increase in femoral offset postoperatively is crucial not only for restoring hip biomechanics and reducing the risk of postoperative complications such as dislocation, but also for improving abductor muscle function. An appropriate femoral offset helps in optimizing the length–tension relationship of the abductor muscles, which can mitigate the risk of abductor weakness or pain postoperatively [23,24]. Failure to adequately restore the femoral offset could lead to abductor dysfunction, resulting in altered gait mechanics and chronic pain, which would compromise the overall success of the surgery [24].

The adjustments in cup inclination and stem anteversion are within acceptable limits, ensuring proper implant positioning. The accurate restoration of cup inclination is particularly important for joint stability and wear reduction [26]. Proper cup inclination minimizes edge loading and uneven wear on the acetabular component, which can prolong the lifespan of the implant [26]. Additionally, maintaining correct stem anteversion is essential for preventing impingement and dislocation, thereby contributing to the long-term success of the total hip arthroplasty [28].

The good fit of the custom stems within the femoral canal, as observed in this study, is a critical factor in ensuring the stability of the implant. Proper stem fit is essential for reducing micromotion, which is a known contributor to aseptic loosening, one of the leading causes of implant failure [29]. Aseptic loosening occurs when micromotion at the bone–implant interface prevents proper osseointegration, leading to implant instability and the potential need for revision surgery [30]. The custom stems used in this study demonstrated a secure fit, particularly in the metaphyseal region, which is crucial for the initial stability of the implant. This stability is vital for promoting bone ingrowth and achieving long-term fixation [16]. Furthermore, the customization of the stem allows for an optimal match to the patient’s unique femoral anatomy, which is especially important in cases of abnormal bone geometry that might not be adequately addressed by standard implants [17]. By reducing the risk of micromotion and enhancing stability, custom stems contribute to the overall success and longevity of the hip arthroplasty, providing patients with improved outcomes and reducing the likelihood of complications over time [31].

Advancements in medical care have resulted in increased life expectancy, with patients now expecting to maintain a high quality of life and independence well into their later years. However, one of the significant challenges with non-cemented femoral stems in THA is the risk of aseptic loosening, which remains a leading cause of prosthesis failure. Custom-made prostheses have shown a reduced risk of this complication, as they can be designed to match the patient’s unique anatomy more accurately than off-the-shelf implants. This customization not only improves the fit and fixation of the implant but also contributes to the long-term stability of the prosthesis, thereby enhancing the overall success rate of the surgery [32]. Aseptic loosening often results from micromotion at the bone–implant interface, which can lead to bone resorption and implant failure. The custom design allows for a more secure fit within the femoral canal, reducing micromotion and promoting better osseointegration, thereby extending the longevity of the implant [29].

To summarize, custom hip implants provide surgeons with an option to reconstruct the hip joint with an amount of accuracy and reliability that can hardly be achieved by using off the shelf implants. Custom implants help minimize common complications associated with THA, which mostly uses standardized implants, such as impingement, dislocation, offset discrepancies, and leg length discrepancies. The reproducibility of the 3D preoperative planning process has been validated in multiple studies, with over 90% accuracy reported when comparing the planned implant positioning to the actual postoperative results. This high level of accuracy underscores the value of custom hip prostheses in achieving consistent and reliable outcomes in THA [33].

Despite the clear clinical advantages, the widespread adoption of custom-made prostheses is limited by several factors. One of the most significant barriers is the cost associated with these implants. The production of custom prostheses involves advanced imaging, precise design, and specialized manufacturing processes, all of which contribute to a higher cost compared to conventional implants. This increased cost can be prohibitive, especially in healthcare systems with limited resources, leading to concerns about the overall cost-effectiveness of custom prostheses [17,19].

Additionally, the time required to produce a custom implant is another critical factor. The process involves several steps, including detailed preoperative planning and the actual fabrication of the implant, which can take several weeks. In cases where immediate surgery is required, the time delay associated with custom prostheses may not be feasible, necessitating the use of readily available conventional implants. This time constraint limits the use of custom prostheses to elective surgeries where there is sufficient time for preoperative planning and implant production [14,15].

Another consideration is the lack of long-term data on the performance of custom-made prostheses compared to conventional implants. While early and mid-term results are promising, with high survivorship and low complication rates, there is still a need for comprehensive long-term studies to confirm these findings. Such data are essential to justify the higher initial costs and to determine whether custom prostheses offer significant long-term benefits over conventional systems, particularly in terms of reducing the need for revision surgeries and improving overall patient satisfaction [19,31,34].

The limitations of this study are that (1) no patient satisfaction was reviewed; (2) no long-term survivorship and complications were evaluated; and (3) the study group was rather small and no control group using conventional THA implants was available.

## 5. Conclusions

The use of custom-made femoral stems in total hip arthroplasty offers distinct advantages, especially in younger patients with altered anatomy and high functional demands. Custom stems allow for precise anatomical reconstruction, reducing the risk of intraoperative complications and postoperative issues such as aseptic loosening and osteolysis. These implants are associated with improved kinematics, enhanced component survival, and a quicker return to high-quality physical activity and life. While custom stems may not be necessary for every patient, they represent a valuable option for those with atypical anatomy, ensuring a tailored approach to THA that optimizes both clinical outcomes and patient satisfaction.

## Figures and Tables

**Table 1:** Patient demographics and baseline characteristics.

		Mean	SD	Min	Max	*p*-Value
Age		55.8	10.2	40.1	74.2	
Hip height	Pre-op	52.4	11.5	26	81	<0.001 *
	Post-op	57.4	10.7	43	85	
	Difference	5	4.8	−9	17	
Femoral offset	Pre-op	39.7	6.6	26	50	<0.001 ^†^
	Post-op	46.2	6.8	34	60	
	Difference	6.5	3.7	0	13	
Femoral anteversion	Pre-op anteversion	16.8	11.1	0	39	0.112 *
	Post-op anteversion	15.1	7.7	3	30	
	Stem anteversion	−0.8	4.2	−9	7	
Cup orientation	Cup inclination	40.0	5.9	30	48	
	Cup anteversion	19.9	13.9	−6	42	

SD = standard deviation. Min = minimum. Max = maximum. * paired T-test. ^†^ Wilcoxon signed-rank test.

**Table 2:** Preoperative and postoperative limb length discrepancy.

		Mean	SD	Min	Max	*p*-Value
Limb Length discrepancy	Pre-op	−5.6	7.5	−24	5	0.003 *
	Post-op	−0.2	4.4	−11	9	

SD = standard deviation. Min = minimum. Max = maximum. * paired T-test.

**Table 3:** Stem Fit Analysis.

		Mean	SD	Min	Max
Stem Fit	Medial	2.0	0.7	1	3
	Lateral	1.0	0.7	0	2
	Anterior	1.1	0.7	0	3
	Posterior	1.4	0.8	0	3

## Data Availability

The original contributions presented in this study are included in the article. Further inquiries can be directed to the corresponding author.

## References

[1] Zacher J., Gursche A. (2003). ‘Hip’ pain. Best Pract. Res. Clin. Rheumatol..

[2] O’Neill T.W., McCabe P.S., McBeth J. (2018). Update on the epidemiology, risk factors and disease outcomes of osteoarthritis. Best Pract. Res. Clin. Rheumatol..

[3] Wyles C.C., Norambuena G.A., Howe B.M., Larson D.R., Levy B.A., Yuan B.J., Trousdale R.T., Sierra R.J. (2017). Cam Deformities and Limited Hip Range of Motion Are Associated with Early Osteoarthritic Changes in Adolescent Athletes: A Prospective Matched Cohort Study. Am. J. Sports Med..

[4] Wyles C.C., Heidenreich M.J., Jeng J., Larson D.R., Trousdale R.T., Sierra R.J. (2017). The John Charnley Award: Redefining the Natural History of Osteoarthritis in Patients with Hip Dysplasia and Impingement. Clin. Orthop. Relat. Res..

[5] Oner A., Koksal A., Sofu H., Aykut U.S., Yıldırım T., Kaygusuz M.A. (2016). The prevalence of femoroacetabular impingement as an aetiologic factor for end-stage degenerative osteoarthritis of the hip joint: Analysis of 1000 cases. Hip Int..

[6] Helgesson L., Johansson P.K., Aurell Y., Tiderius C.J., Kärrholm J., Riad J. (2018). Early osteoarthritis after slipped capital femoral epiphysis. Acta Orthop..

[7] Stibolt R.D., Jr Patel H.A., Huntley S.R., Lehtonen E.J., Shah A.B., Naranje S.M. (2018). Total hip arthroplasty for posttraumatic osteoarthritis following acetabular fracture: A systematic review of characteristics, outcomes, and complications. Chin. J. Traumatol..

[8] Roth A., Beckmann J., Bohndorf K., Fischer A., Heiß C., Kenn W., Jäger M., Maus U., Nöth U., Peters K.M. (2016). S3-Guideline non-traumatic adult femoral head necrosis. Arch. Orthop. Trauma. Surg..

[9] Learmonth I.D., Young C., Rorabeck C. (2007). The operation of the century: Total hip replacement. Lancet.

[10] Liu X.W., Zi Y., Xiang L.B., Wang Y. (2015). Total hip arthroplasty: Areview of advances, advantages and limitations. Int. J. Clin. Exp. Med..

[11] McAuley J.P., Szuszczewicz E.S., Young A., Engh C.A. (2004). Total hip arthroplasty in patients 50 years and younger. Clin. Orthop. Relat. Res..

[12] Eskelinen A., Remes V., Helenius I., Pulkkinen P., Nevalainen J., Paavolainen P. (2005). Total hip arthroplasty for primary osteoarthrosis in younger patients in the Finnish arthroplasty register. 4,661 primary replacements followed for 0–22 years. Acta Orthop..

[13] Laine H.J., Puolakka T.J., Moilanen T., Pajamäki K.J., Wirta J., Lehto M.U. (2000). The effects of cementless femoral stem shape and proximal surface texture on ‘fit-and-fill’ characteristics and on bone remodeling. Int. Orthop..

[14] Maggs J., Wilson M. (2017). The Relative Merits of Cemented and Uncemented Prostheses in Total Hip Arthroplasty. Indian. J. Orthop..

[15] Watts C.D., Abdel M.P., Lewallen D.G., Berry D.J., Hanssen A.D. (2015). Increased risk of periprosthetic femur fractures associated with a unique cementless stem design. Clin. Orthop. Relat. Res..

[16] Mirza S.B., Dunlop D.G., Panesar S.S., Naqvi S.G., Gangoo S., Salih S. (2010). Basic science considerations in primary total hip replacement arthroplasty. Open Orthop. J..

[17] Argenson J.N., Flecher X., Parratte S., Aubaniac J.M. (2007). Anatomy of the dysplastic hip and consequences for total hip arthroplasty. Clin. Orthop. Relat. Res..

[18] Jenny J.Y., Barbe B. (2012). Small differences between anatomical and mechanical sagittal femur axes: A radiological and navigated study of 50 patients. Arch. Orthop. Trauma. Surg..

[19] Flecher X., Pearce O., Parratte S., Aubaniac J.M., Argenson J.N. (2010). Custom cementless stem improves hip function in young patients at 15-year followup. Clin. Orthop. Relat. Res..

[20] Wettstein M., Mouhsine E., Aubaniac J.M., Audigé L., Ollivier M., Leyvraz P.F., Argenson J.N. (2023). The torsion of the proximal femur in cementless total hip arthroplasty: A 3-dimensional evaluation. Hip Int..

[21] Lazennec J.Y., Brusson A., Dominique F., Rousseau M.A., Pour A.E. (2015). Offset and anteversion reconstruction after cemented and uncemented total hip arthroplasty: An evaluation with the low-dose EOS system comparing two- and three-dimensional imaging. Int. Orthop..

[22] Steppacher S.D., Tannast M., Werlen S., Siebenrock K.A. (2008). Femoral morphology differs between deficient and excessive acetabular coverage. Clin. Orthop. Relat. Res..

[23] Mahmood S.S., Mukka S.S., Crnalic S., Wretenberg P., Sayed-Noor A.S. (2016). Association between changes in global femoral offset after total hip arthroplasty and function, quality of life, and abductor muscle strength. A prospective cohort study of 222 patients. Acta Orthop..

[24] Rüdiger H.A., Guillemin M., Latypova A., Terrier A. (2017). Effect of changes of femoral offset on abductor and joint reaction forces in total hip arthroplasty. Arch. Orthop. Trauma. Surg..

[25] Sariali E., Klouche S., Mamoudy P. (2012). Investigation into three dimensional hip anatomy in anterior dislocation after THA. Influence of the position of the hip rotation centre. Clin. Biomech..

[26] Tsai T.Y., Dimitriou D., Li G., Kwon Y.M. (2014). Does total hip arthroplasty restore native hip anatomy? three-dimensional reconstruction analysis. Int. Orthop..

[27] Dessyn E., Flecher X., Parratte S., Ollivier M., Argenson J.N. (2019). A 20-year follow-up evaluation of total hip arthroplasty in patients younger than 50 using a custom cementless stem. Hip Int..

[28] Kim Y.H., Oh S.H., Kim J.S. (2003). Primary total hip arthroplasty with a second-generation cementless total hip prosthesis in patients younger than fifty years of age. J. Bone Jt. Surg. Am..

[29] Streit M.R., Haeussler D., Bruckner T., Proctor T., Innmann M.M., Merle C., Gotterbarm T., Weiss S. (2016). Early Migration Predicts Aseptic Loosening of Cementless Femoral Stems: A Long-term Study. Clin. Orthop. Relat. Res..

[30] Brown T.E., Larson B., Shen F., Moskal J.T. (2002). Thigh pain after cementless total hip arthroplasty: Evaluation and management. J. Am. Acad. Orthop. Surg..

[31] Wettstein M., Mouhsine E., Argenson J.N., Rubin P.J., Aubaniac J.M., Leyvraz P.F. (2005). Three-dimensional computed cementless custom femoral stems in young patients: Midterm followup. Clin. Orthop. Relat. Res..

[32] Tikhilov R.M., Dzhavadov A.A., Kovalenko A.N., Bilyk S.S., Denisov A.O., Shubnyakov I.I. (2022). Standard Versus Custom-Made Acetabular Implants in Revision Total Hip Arthroplasty. J. Arthroplast..

[33] Tostain O., Debuyzer E., Benad K., Putman S., Pierache A., Girard J., Pasquier G. (2019). Ten-year outcomes of cementless anatomical femoral implants after 3D computed tomography planning. Follow-up note. Orthop. Traumatol. Surg. Res..

[34] Faldini C., Miscione M.T., Chehrassan M., Acri F., Pungetti C., d’Amato M., Luciani D., Giannini S. (2011). Congenital hip dysplasia treated by total hip arthroplasty using cementless tapered stem in patients younger than 50 years old: Results after 12-years follow-up. J. Orthop. Traumatol..

